# Treatment of the Oxalate of Lime Deposits

**Published:** 1847-05

**Authors:** 


*Treatment of the Oxalate of Lime Deposits.*—Although there can be little doubt that oxalic acid is generated in the urine by the putrefactive decomposition, and often occurs subsequent to secretion in a manner totally independent of disease, yet it is also certain that this decomposition frequently results from an essential vice of secretion. The morbid conditions which give rise to this change are not yet known; but one thing is well ascertained, that in every instance of the kind there is frequent desire to pass water, pain in passing it, and that the secretion is commonly loaded with epithelium. These phenomena proclaim the existence of irritation of the mucous membrane. Sometimes the crystals of oxalate of lime, like those of uric acid, cohere in the calyces and infundibula, forming calculi, which produce paroxysms of nephritic colic by their descent into the bladder. In such cases inflammation of the lining membrane may be mechanically produced; but it is doubtful whether the symptoms of mucous irritation which usually accompany the oxalate of lime deposit are due to the irritating contact of the sharp crystals. Whatever is the cause of the mucous irritation, it constitutes the lesion which, in oxalate of lime diathesis, you are especially called on to remove; and its successful treatment requires no little delicacy in the application of therapeutical agents. Unlike acute mucous irritation, depletion and emollients will act in this irritation injuriously, if employed in the first instance; you must have recourse to tonics immediately, such as the mineral acids, vegetable bitter astringents, &c.; and having employed those means for some time, you will then find the greatest benefit from alkalies largely diluted.

It will be often necessary to alternate these methods of treatment for a considerable period, but you will generally find that ultimate benefit will be derived from steady persistence in their use. The form of tonic mixture which I usually employ in these cases, is the following:

℞. Infusi cascarillæ, ℥vj.

Nitratis potassæ, ʒj.

Acidi nitrici diluti, ʒiss.

Tincturæ opii, ʒj.

M. Sumat cochlearia quo ampla ter in die.

*Ranking's Abstract.*

